# The effect of an intracerebroventricular injection of metformin or AICAR on the plasma concentrations of melatonin in the ewe: potential involvement of AMPK?

**DOI:** 10.1186/1471-2202-12-76

**Published:** 2011-07-29

**Authors:** Jean-Baptiste Menassol, Claire Tautou, Armelle Collet, Didier Chesneau, Didier Lomet, Joëlle Dupont, Benoît Malpaux, Rex J Scaramuzzi

**Affiliations:** 1 Physiologie de la Reproduction et des Comportements UMR85, INRA, Nouzilly F-37380, France; 2 Physiologie de la Reproduction et des Comportements UMR6175, INRA, Nouzilly F-37380, France; 3 Université François Rabelais de Tours, Tours F-37041, France; 4 IFCE, Nouzilly F-37380, France; 5 Department of Veterinary Basic Sciences, Royal Veterinary College, Herts, UK

## Abstract

**Background:**

It is now widely accepted that AMP-activated protein kinase (AMPK) is a critical regulator of energy homeostasis. Recently, it has been shown to regulate circadian clocks. In seasonal breeding species such as sheep, the circadian clock controls the secretion of an endogenous rhythm of melatonin and, as a consequence, is probably involved in the generation of seasonal rhythms of reproduction. Considering this, we identified the presence of the subunits of AMPK in different hypothalamic nuclei involved in the pre- and post-pineal pathways that control seasonality of reproduction in the ewe and we investigated if the intracerebroventricular (i.c.v.) injection of two activators of AMPK, metformin and AICAR, affected the circadian rhythm of melatonin in ewes that were housed in constant darkness. In parallel the secretion of insulin was monitored as a peripheral metabolic marker. We also investigated the effects of i.c.v. AICAR on the phosphorylation of AMPK and acetyl-CoA carboxylase (ACC), a downstream target of AMPK, in brain structures along the photoneuroendocrine pathway to the pineal gland.

**Results:**

All the subunits of AMPK that we studied were identified in all brain areas that were dissected but with some differences in their level of expression among structures. Metformin and AICAR both reduced (p < 0.001 and p < 0.01 respectively) the amplitude of the circadian rhythm of melatonin secretion independently of insulin secretion. The i.c.v. injection of AICAR only tended (p = 0.1) to increase the levels of phosphorylated AMPK in the paraventricular nucleus but significantly increased the levels of phosphorylated ACC in the paraventricular nucleus (p < 0.001) and in the pineal gland (p < 0.05).

**Conclusions:**

Taken together, these results suggest a potential role for AMPK on the secretion of melatonin probably acting trough the paraventricular nucleus and/or directly in the pineal gland. We conclude that AMPK may act as a metabolic cue to modulate the rhythm of melatonin secretion.

## Background

The serine-threonine kinase, AMPK integrates energy homeostasis at both cellular [1] and whole-body levels [2]. It exists as a heterotrimeric complex of a catalytic α subunit and two regulatory β and γ subunits. In mammals there are two or three isoforms of each subunit (α1, α2, β1, β2, γ1, γ2, γ3), encoded by different genes, allowing for a variety of heterotrimeric combinations [3]. The treatment with pharmacological agents such as metformin and 5-aminoimidazole-4-carboxamide-1-β-d-ribofuranoside (AICAR) activates the AMPK complex both *in vitro *and *in vivo *[4-6]. This mechanism is central in the action of metformin when used as a insulin-sensitizing agent to treat type II diabetes [7]. Recent evidence points to an emerging role for AMPK as a critical element linking metabolism and reproduction [8]. Indeed, we demonstrated that treatment with either metformin or AICAR significantly inhibited the release of GnRH from immortalised GnRH neurones (GT1-7 cells) and *in vivo*, it shortened the inter-oestrus interval in mice [6]. In sheep as in all seasonal breeding species, the daily rhythm of melatonin secretion is the critical element mediating the synchronising effect of photoperiod on reproduction. This rhythm is endogenously controlled by the activity of the master circadian pacemaker located in the suprachiasmatic nucleus [9]. However, how this endogenous rhythm of melatonin affects the seasonal activity of the GnRH/LH/gonadal axis remains unclear.

Um *et al*. [10] demonstrated that the peripheral activation of AMPK with metformin induced phase-shifts in some of the core clock proteins thus suggesting a role for AMPK in the control of circadian rhythms. In support of these observations we have identified AMPK in the sheep brain and we have tested, the effects of i.c.v. injections of metformin or AICAR on the secretion of melatonin in ewes housed in constant darkness. In parallel, because of the functional interrelationships between melatonin and insulin [11,12], we also monitored the secretion of insulin as a potential metabolic marker of peripheral AMPK activation [13]. Our results suggest that i.c.v. injections of metformin and AICAR both affect the amplitude of the circadian rhythm of melatonin secretion without altering the patterns of plasma insulin secretion.

## Methods

### Hormones and reagents

The metformin and AICAR used for the i.c.v. injections were obtained from Sigma (Saint Quentin Fallavier, France). The dose injected by means of an intracerebroventricular catheter, was 128 mM for metformin (adapted to sheep from [14]) and 12 mM for AICAR (similar to [6]) in a total volume of 50 μl. Untreated control ewes were injected with the same volume of an artificial cerebrospinal fluid (CSF) solution, prepared in the laboratory. All solutions had an equivalent osmolarity (≈ 305 mOsm).

### Antibodies

Rabbit polyclonal antibodies to phospho-AMPKα Thr172, AMPKα1/2, AMPKβ1/β2, AMPKγ2, phospho-acetyl-CoA carboxylase (ACC) Ser79 and ACC were purchased from New England Biolabs Inc. (Beverly, MA, USA). A mouse monoclonal antibody to vinculin was obtained from Sigma (Saint Quentin Fallavier, France) and used as an internal standard.

### Animals and experimental designs

Nineteen multiparous ewes were surgically fitted with cannulae in the anterodorsal region of their third ventricles [15]. Animals were housed in individual pens in a light-sealed room under a lightning program mimicking a long-day photoperiod (LP) (16 hours of light/day). All ewes had free access to food, water and mineral licks.

**Figure 1:**
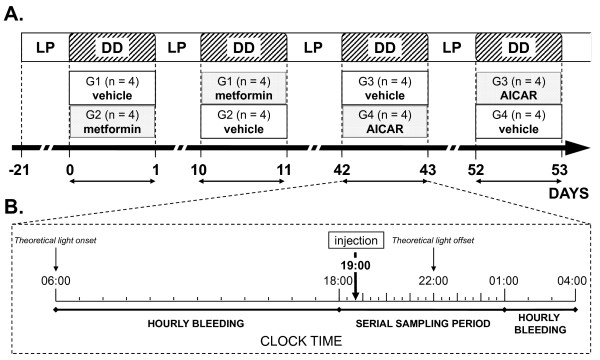
**Experimental design for the first experiment**. **(A) **Schematic representation of the cross-over experimental design. Photoperiodic treatments are given in the upper part. 3 weeks before the first day of injection (D0) the ewes were housed under LP. During each day of injection (D0 and D10 for metformin; D42 and D52 for AICAR) LP was replaced by DD. **(B) **Bleeding protocol operated during DD, each thick depicts one sample. Thick arrow depict the clock time for the administration of the injection (19:00). Serial sampling period began 1 hr before injection and lasted for 7 hrs (from 17:00 to 00:00 during the injection of metformin and adjusted from 18:00 to 01:00 for the injection of AICAR). Thin arrows depict theoretical lightning transitions. See text for more details.

In the first experiment, two groups of ewes (G1, G2, n = 4) balanced for body condition score and live weight were used in a cross-over design so that each group received in random order 9 days apart (day 0 or D0 and D10), either artificial CSF or metformin. Forty days later, groups were re-balanced for their night-time plasma concentrations of melatonin (G3, G4, n = 4), and the experiment was repeated using AICAR with the same cross-over design (D42 and D52) (Figure 1A). For each i.c.v. injection, the ewes were fitted with an indwelling catheter in the left jugular vein and housed in constant darkness (DD) for one whole light/dark (L/D) cycle. The ewes were bled every 60 min for 22 hrs, from 06:00 to 04:00 the next day, with a period of more intensive blood sampling (every 20 min) around the time of the i.c.v. injection at 19:00 (Figure 1B). Blood (3 ml) was collected into heparinised tubes and the plasma was separated by centrifugation (3700 g, 20 min, 4°C) and stored at -20°C.

For the second experiment, two groups of ewes balanced as for experiment 1, were injected with either AICAR (n = 6) or artificial CSF (n = 5). One hour after injection the animals were slaughtered by decapitation, the brain was removed quickly and frozen in isopentane (SDS, Val De Rueil, France). The pituitary (PIT) and pineal (PIN) glands were immediately dissected, frozen in liquid nitrogen and stored at -80°C. Frontal sections (1 mm thick) were sliced from the diencephalon and other structures of interest were dissected using a sterile scalpel, all were frozen in liquid nitrogen and stored at -80°C. The hypothalamic structures of interest (suprachiasmatic nucleus [SCN], arcuate nucleus [ARC], median eminence [ME], paraventricular nucleus [PVN], dorsomedial hypothalamic nucleus [DMH], tanycytes [TAN] and premammilary hypothalamic nucleus [PMH]) were identified using sheep brain atlases [16,17].

All these procedures were approved by the French Agricultural and Scientific Research Agencies and conducted in accordance with the EU guidelines for Care and Use of Agricultural Animals in Agricultural Research and Teaching.

### Western-blotting

Western-blotting procedures were conducted as previously described [6] using 60 μg of protein per sample. The data for the phosphorylation of AMPK and ACC are expressed as the ratio of phosphorylated AMPK to total AMPK and phosphorylated ACC to total ACC respectively. The data for the AMPK subunits have been normalised against an internal standard (vinculin).

### Assays

Melatonin was assayed using a radioimmunoassay (RIA) described by Fraser *et al*. [18], with an antibody raised by Tillet *et al*. [19]. The assay sensitivity was 4 pg/mL, the intra-assay coefficient of variation (CV) ranged between 9-14% and the inter-assay CV was 12%. Insulin was also assayed using a RIA that was developed in our laboratory. Plasma concentrations of insulin were determined in duplicate 100 μL samples. The assay buffer was 0.025 M barbital buffer containing 3% bovine serum albumin (Sigma, Saint Quentin Fallavier, France). Plasma samples were incubated (24 hrs at 4°C) with 300 μL of the primary antibody (anti-porcine insulin antibody raised in guinea pig, final dilution 1/80000; Sigma, Saint Quentin Fallavier, France) added with 0.6 μL of Triton X-100 and 0.5 μL of normal rabbit serum. Samples were then incubated (24 hrs) with 20,000 cpm of iodinated porcine insulin. The next day, samples were incubated for 48 hrs at 4°C with 2 mL of the secondary antibody (anti-guinea pig IgG raised in horses, final dilution 1/333), 4 g/L of CaCl_2 _and 30 g/L of polyethylene glycol. Tubes were then centrifuged (3,000 g for 30 min at 4°C), the supernatant was removed, and the radioactivity in the precipitate was counted with a Wallac Wizard gamma counter (PerkinElmer Inc., Waltham, Massachusetts, USA) for 1 min. The sensitivity of the assay was 0.95 ± 0.06 ng/mL and the intra-assay CV was 12%.

### Data and Statistical analysis

Melatonin profiles were analysed by comparing the timing of resumption and the amplitude, of the circadian rhythm of melatonin secretion. These criteria were determined using the method of Malpaux *et al*. [20]. Statistical analyses were performed using the statistical software package, R [21]. Data for insulin were analysed using a mixed-effects model and other data, after log transformation for normalization, were analysed using an analysis of variance. All results are presented as means ± sem. Differences were considered statistically significant whenever p < 0.05.

## Results

**Figure 2:**
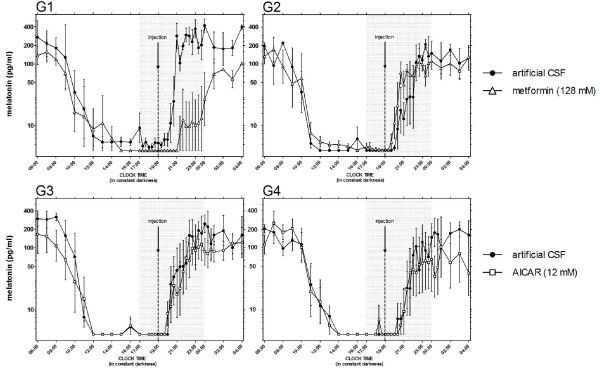
**The monitoring of the endogenous rhythm of secretion of melatonin**. The data show the concentration of plasma melatonin for each group separately (G1 to G4). Animals were housed in constant darkness and given an i.c.v. injection of either metformin (**G1 & G2**; open triangle) or AICAR (**G3 & G4**; open square) or artificial CSF as a control vehicle (closed circle). Injections were performed nine days apart and in a random order for each group (see text and Figure 1 for more details about the order of injection for each group). The arrows depict the time of injection and shaded area corresponds to the period of increased sampling frequency. Note the log-scale y-axis.

**Figure 3:**
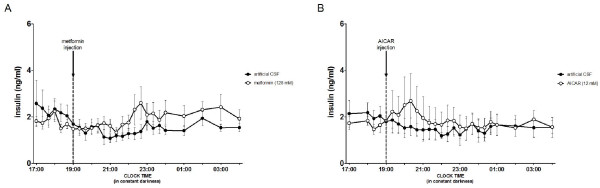
**The monitoring of the secretion of plasma insulin**. The data show the concentration of plasma insulin in two groups of ewes housed in constant darkness and given an i.c.v. injection of either **(A) **metformin or **(B) **AICAR (open circle) or a synthetic CSF as a control vehicle (closed circle). The arrows depict the time of injection.

The amplitude of the endogenous rhythm of melatonin was reduced significantly by both metformin (p < 0.001) and AICAR (p < 0.01) (Figure 2) but the timing of resumption was not affected by either (data not shown). After each i.c.v. injection of metformin at D0 and D10 (Figure 2A), the amplitude of the endogenous rhythm of melatonin was reduced when compared to controls (p < 0.001). This was also the case between D0 and D10 for the ewes in G1 (p < 0.001), that is those ewes injected first with the vehicle solution, but not for the ewes in G2 that is those ewes injected first with metformin. After injection of AICAR (Figure 2B), the amplitude of the endogenous rhythm of melatonin was reduced at D42 compared to controls (p < 0.001) but not at D52. However, when compared between D42 and D52, the amplitude of the endogenous rhythm of melatonin was consistently reduced after the injection of AICAR in both groups (p < 0.05). There was no significant effect of metformin or AICAR on the concentrations of insulin (Figure 3).

**Figure 4:**
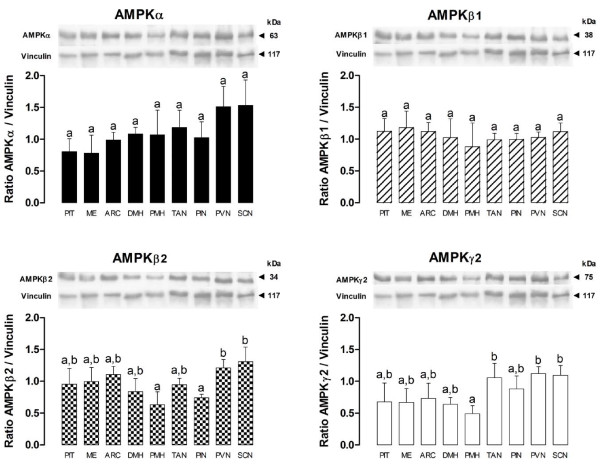
**Western-blotting quantification of the AMPK subunits α, β1, β2 and γ2 in pituitary gland (PIT), median eminence (ME), arcuate nucleus (ARC), dorsomedial and premammilary hypothalamus (DMH, PMH), tanycytes (TAN), pineal gland (PIN) and paraventricular and suprachiasmatic nuclei (PVN, SCN) of the sheep brain**. Different letters indicate a stastistically significant difference (p < 0.05).

**Figure 5:**
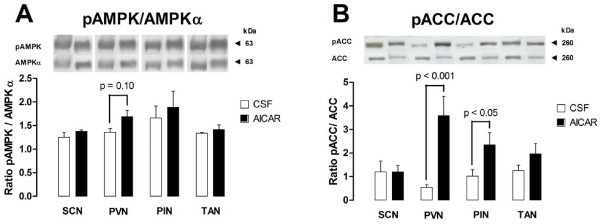
**Western-blotting quantification of the phosphorylation rate of AMPK and ACC after i.c.v. injection of AICAR**. **(A) **The ratio of phosphorylated AMPK at Thr172 to total AMPK following an i.c.v. injection of a synthetic CSF vehicle or AICAR. **(B) **The ratio of phosphorylated ACC at Ser79 to total ACC following an i.c.v. injection of a synthetic CSF vehicle or AICAR.

The results of experiment 2 show that the expression levels of the AMPK subunits (Figure 4) were different among hypothalamic structures. The expression of the β2 subunit was lower in the PMH/PIN when compared to the PVN/SCN (p < 0.05) and the expression of the γ2 subunit was lower in the PMH when compared to the PVN/SCN/TAN (p < 0.05). After i.c.v. injection of AICAR, the phosphorylation rate of AMPK tended (p = 0.1) to be higher only in the PVN (Figure 5A) but the phosphorylation rate of ACC at Ser79 was significantly higher in both the PVN (p < 0.001) and PIN (p < 0.05) (Figure 5B).

## Discussion

The hypothesis tested in this study, developed from the findings of Um et al. [10], was that central activation of AMPK could disrupt the circadian machinery located in the SCN and thus, alter the endogenous timing of the resumption of melatonin secretion by the pineal gland. In our study, such an effect was not observed after i.c.v. treatment with metformin or AICAR, both known activators of AMPK. Surprisingly we found that another component of the endogenous rhythm of melatonin, its amplitude, was altered by these compounds.

There are three main possibilities to account for the rejection of our hypothesis. First, the i.c.v. treatment with either metformin or AICAR was ineffective and did not stimulate AMPK in the hypothalamic structures we examined. Indeed, the similarities of the effects between the i.c.v. injections of both drugs on the amplitude of the rhythm of melatonin secretion suggest that they might be mediated by the activation of AMPK, as a common element along their distinct signalling pathways [22]. However, we do not exclude possible AMPK-independent effects [23]. Second, the treatment effectively activated AMPK within the SCN, but in DD when the SCN is released from entrainment by the prevailing LP produced conflicting signals because of the persistence of entrainment by the preceding LP [24] and the expected SCN-driven phase shift in the resumption of melatonin secretion. Third, the treatment effectively activated AMPK but not within the SCN or without triggering phase-shifts in the master circadian machinery perhaps because of functional differences [25] between the master circadian clock and the peripheral-slaves clock that was studied by Um et al. [10].

The biological relevance of the lowering effects of both metformin and AICAR on the amplitude of the endogenous rhythm of melatonin is difficult to interpret. Indeed, in sheep as in most photoperiodic species, the duration of melatonin secretion is the critical feature of the rhythm of melatonin secretion that transmits photoperiodic information to the reproductive axis [26]; the role played by the amplitude, the other main feature of this rhythm, remains controversial [27-29]. Some authors have suggested that the amplitude of the melatonin rhythm acts as a potent integrator of environmental factors, other than photoperiod for example, temperature and the food supply (for review see [30]). In relation to this theory, a complementary study (Menassol *et al*., unpublished data) has established that feed restriction can alter the seasonal reproductive patterns of the Île-de-France ewe as has been reported in a previous study on sheep [31], and that this alteration is associated with lower concentrations of plasma melatonin. Therefore we speculate that the central activation of AMPK is involved in the integration of metabolic-related cues that alter the amplitude of the rhythm of melatonin secretion. We speculate further that this effect is mediated by neuropeptides or peptidergic hormones that regulate the amplitude of the rhythm of melatonin secretion such as Vasoactive Intestinal Peptide (VIP), Pituitary Adenylate Cyclase Activating Peptide (PACAP) or Vasopressin (VP) (for review see [32]) and particularly Neuropeptide Y (NPY) which is activated by AMPK [33].

The effect of metformin and AICAR on the amplitude of the rhythm of melatonin secretion was not associated with immediate changes in insulin secretion and thus, was not attributable to the interrelationship that exists between these two hormones [11,12,34]. Interestingly, some studies have reported a decrease in melatonin concentrations associated with the development of diabetes [35,36]. Thus, assuming that metformin crosses the blood-brain barrier, one can ask if therapeutic treatment with metformin could not have unreported side-effects on the secretion of melatonin.

The characterization of the expression of AMPK within the hypothalamus, pituitary and pineal glands of the ewes suggests a potentially wide range of action for metformin and AICAR since all subunits were identified in each of the structures that we examined. The differences among structures in the level of expression of the β2 and γ2 subunits may indicate structural specificities in the sensitivity or functionality of the AMPK complex [2]. Indeed, our investigation of the potential central sites of actions of the AMPK complex, point preferentially to the PVN as a structure of particular interest because some of its neurons that express melatonin-regulating peptides project to the pineal gland [32]. In this particular structure, the direct assessment of the phosphorylation rate of AMPK only reveals a trend toward significance (p = 0.1) for a greater activation after i.c.v. injection of AICAR. However the indirect measurement of AMPK activity, trough the assessment of the phosphorylation rate of its downstream target ACC [37,38], shows that AICAR treatment significantly inhibited ACC activity in both the paraventricular nucleus (p < 0.001) and the pineal gland (p < 0.05). Therefore the PVN is strongly suggested as a key structure that mediates metabolic influences on the rhythm of secretion of melatonin. Moreover, it seems that direct effects of AMPK on the pineal gland activity itself can not be excluded.

In this experiment, the failure to demonstrate significant direct activation of the AMPK complex following treatment with AICAR might be explained by the time elapsed between tissue fixation and injection (1 hr) and the possible action of endogenous phosphatases [39].

## Conclusion

To conclude, the i.c.v. administration of AMPK-activators had an unexpected insulin-independent effect on the amplitude of the endogenous rhythm of secretion of melatonin that retrospectively, might have also been observed in natural photoperiodic conditions, on the daily pattern of melatonin secretion. This effect probably involves the AMPK system trough the paraventricular nucleus and/or directly within the pineal gland. This mechanism remains to be precisely determined but we suggest that it involves the integration of metabolic-related cues with the photoperiodic signal that synchronizes the annual rhythm of reproduction in sheep.

## Competing interests

The authors declare that they have no competing interests.

## Authors' contributions

The authors contributed equally to this study. JBM conceived the study, carried out the experiment, collected data, and drafted the manuscript. CT collected data and performed the melatonin assays. AC and DC followed the experiment and performed melatonin assays. DL performed the injection of drugs and blood sampling. JD, BM and RJS designed the study. JBM, CT, JD, BM and RJS were involved in the analysis and interpretation of the data. JBM and JD revised the manuscript. All authors read and approved the final manuscript.
